# A case of COVID-19-associated rhino-orbito-cerebral mucormycosis caused by *Apophysomyces variabilis* with a review of the literature

**DOI:** 10.3389/fcimb.2022.898477

**Published:** 2022-10-14

**Authors:** Mahzad Erami, Hossein Mirhendi, Mansooreh Momen-Heravi, Seyed Jamal Hashemi Hezaveh, Amir Hossein Ahsaniarani, Seddighe Sadat Sabet, Shima Aboutalebian

**Affiliations:** ^1^ Department of Medical Parasitology and Mycology, School of Public Health, Tehran University of Medical Sciences, Tehran, Iran; ^2^ Infectious Disease Research Center, Kashan University of Medical Sciences, Kashan, Iran; ^3^ Department of Medical Parasitology and Mycology, School of Medicine, Isfahan University of Medical Sciences, Isfahan, Iran; ^4^ Mycology Reference Laboratory, Research Core Facilities Laboratory, Isfahan University of Medical Sciences, Isfahan, Iran; ^5^ Department Otorhinolaryngology, School of Medicine, Matini Hospital, Kashan University of Medical Sciences, Kashan, Iran; ^6^ Department of Pharmaceutical, Kashan University of Medical Sciences, Kashan, Iran

**Keywords:** mucormycosis, *apophysomyces variabilis*, COVID-19, immunocompetent patient, invasive fungal infection, antifungal susceptibility testing

## Abstract

A fatal case of COVID-19-associated mucormycosis (CAM) affected a 40-year-old woman who was initially admitted to our hospital due to a SARS-CoV-2 infection. Her clinical condition worsened, and she finally died because of respiratory failure, hemodynamic instability, and mucormycosis with invasion into the orbit and probably the brain. According to DNA sequence analysis of the fungus isolated from the patient, *Apophysomyces variabilis* was involved. This is the first published case of CAM and the third case of mucormycosis due to this mold.

## Introduction

Mucormycosis (zygomycosis) is a disease caused by an opportunistic infection in humans and animals by members of the order Mucorales (Kwon-Chung, 2012; Binder et al., 2014). Mucormycosis is subdivided into the rhinocerebral, pulmonary, cutaneous, gastrointestinal, and disseminated types according to the distribution of lesions (Roden et al., 2005; Skiada et al., 2011) and mainly occurs in immunosuppressed hosts, including those with uncontrolled diabetes mellitus, hematological malignancies, transplant recipients, and patients receiving corticosteroid therapy (Farmakiotis and Kontoyiannis, 2016). These infections have the capability of angio-invasion, inflicting vasculitis and thrombosis of vessels, which may result in massive areas of infarction and necrosis (Ibrahim et al., 2012). Regardless of its type, zygomycosis is an aggressive, life-threatening disease. As stated by the United States Centers for Disease Control and Prevention (CDC), this is an infrequent illness with an infection rate of <1% worldwide and with a 50% mortality (Dannaoui and Lackner, 2020); if left untreated, the mortality rate could be above 90% (Divakar, 2021).

Most severe cases of COVID-19 suffer from low oxygen levels, high iron levels, a hyperglycemic state, acidosis, and lowered phagocytic activity. These conditions facilitate opportunistic fungal infections, such as mucormycosis (Singh et al., 2021). Recently, there has been a dramatic increase in COVID-19-associated mucormycosis (CAM) cases, driven especially by low- and middle-income countries and most extensively reported in India. The mortality rate varies between 14% and 70%, depending on the site of infection (Patel et al., 2020).

Early diagnosis and treatment are critical to reduce mortality in patients with zygomycosis (Binder et al., 2014). In addition, precise species identification of the fungus involved is critical, not only to ensure accurate diagnosis but also for developing species-specific antifungal drugs, as well as to improve knowledge of the epidemiology of the disease. Various organisms have been implicated as etiological agents of mucormycosis, most commonly *Rhizopus*. Other etiological agents include *Mucor*, *Rhizomucor*, *Lichtheimia* (previously known as *Absidia*), *Apophysomyces*, *Saksenaea*, *Cunninghamella*, *Syncephalastrum*, and *Cokeromyces* (Gomes et al., 2011).

Here, a fatal case of rhino-orbito-cerebral mucormycosis (ROCM) due to *Apophysomyces variabilis* in a 40-year-old woman with COVID-19 is reported. To the authors’ knowledge, this is the first case of CAM caused by *A. variabilis* in Iran. To better understand the clinical characteristics of the cases of ROCM due to non-*Rhizopus* Mucorales, we reviewed all published cases of ROCM that were supported by data from molecular species identification.

## Case report

**Figure 1:**
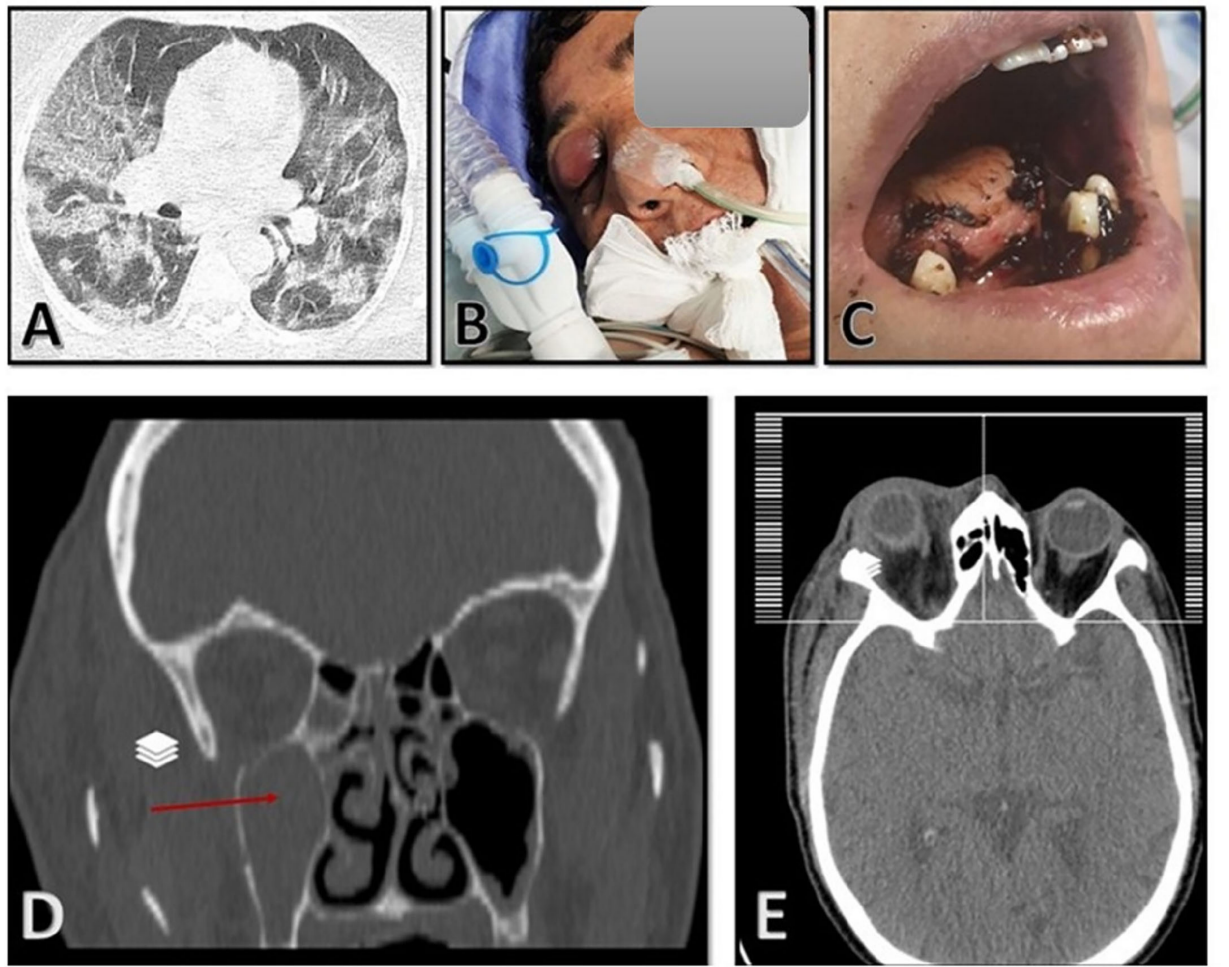
**(A)** Chest computed tomography (CT) scan with multifocal bilateral mixed ground-glass opacity and consolidation, including central and peripheral distribution, and bilateral pleural effusion. **(B, C)** Swelling of the right eye and deep necrotic ulcer in the palate. **(D, E)** Paranasal and orbital CT scan demonstrated opacification and air-fluid level in the bilateral sphenoid and ethmoid sinuses and the right maxilla along with mucosal thickening in the left maxilla and fat haziness in the right extraconal and intraconal locations.

**Figure 2:**
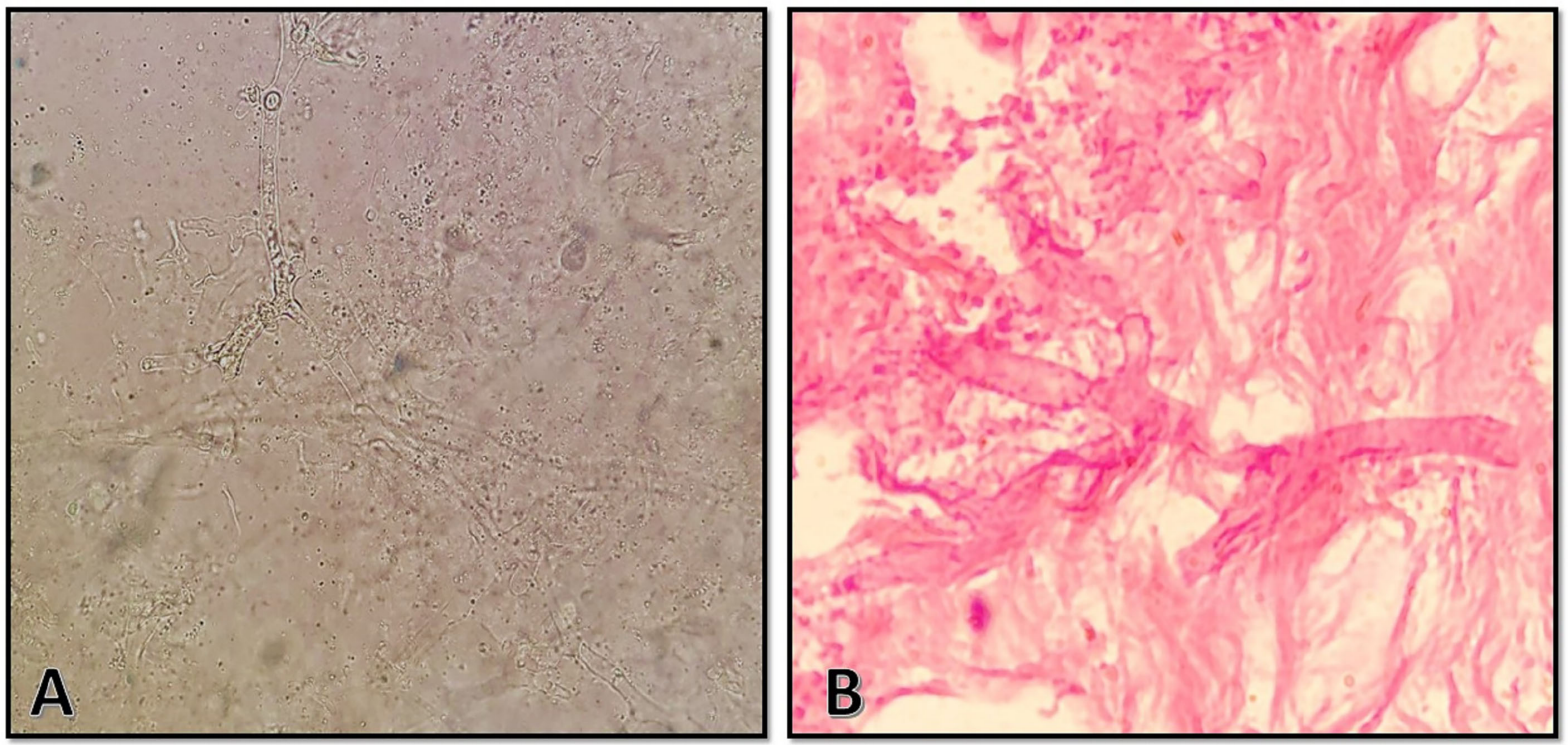
**(A)** Direct microscopic examination using 10% potassium hydroxide revealed broad, aseptate hyaline hyphae with wide-angle branching. **(B)** Histopathologic examination with hematoxylin and eosin stain showing abundance in granulation tissue with foreign-body reaction and broad, aseptate hyphae in the intravascular space with wide-angle branching.

On 11 September 2021, a 40-year-old Afghan woman was hospitalized in Shahid Beheshti Hospital, Kashan, Iran, due to COVID-19. She had a fever, dry cough, dyspnea, hemoptysis, myalgia, and severe weakness, which had been ongoing for 5 days. Physical examination revealed a body temperature of 38.7°C, a blood pressure of 100/80 mm Hg, a respiratory rate of 26 breaths per minute, and an oxygen saturation of 90% on ambient air. Laboratory examination revealed leukopenia, lymphopenia, and a significant increase in C-reactive protein (CRP), and the patient underwent oxygen supplementation therapy. Nasopharyngeal and oropharyngeal swabs were obtained and subject to a real-time reverse transcriptase polymerase chain reaction test using the Light Cycler 96-well system (Roche, Germany) for SARS-CoV-2 targeting N and RdRp genes (Pishtaz Teb, Tehran, Iran) (Fakhim et al., 2021). The sample was positive with a cycle threshold value of 25, indicating ongoing viral infection. Cultures of urine and blood were unremarkable. A computed tomography (CT) scan of the chest demonstrated multifocal bilateral mixed ground-glass opacity and consolidation, including central and peripheral distribution, and bilateral pleural effusion (**Figure 1A**). The patient was started on remdesivir (100 mg), dexamethasone (8 mg intravenously/twice daily and prednisolone (125 mg/day), and anticoagulants (heparin (QZD)). Because of increased hypoxia and progressive reduction in oxygen saturation (≈45%), the patient became severely ill and underwent mechanical ventilation, and treatment with intravenous tocilizumab (8 mg/kg) was started. After 7 days of hospitalization, the patient developed right-side periorbital edema, proptosis of the right globe, necrosis of the hard palate, and nasal bleeding (**Figures 1B, C**). A paranasal and orbital CT scan demonstrated opacification and an air-fluid level in the bilateral sphenoid and ethmoid sinuses and the right maxilla along with mucosal thickening in the left maxilla and fat haziness in the right extra- and intraconal locations (**Figures 1D, E**). Samples from wounds, tissue necrosis, and bloody secretions of the eye were taken and subjected to direct examination by microscopy using a potassium hydroxide preparation (**Figure 2A**), fungal culture, and histopathology (**Figure 2B**). The condition was diagnosed as a probable invasive mucormycosis. Intravenous liposomal amphotericin B 5 mg/kg/day was initiated. However, the patient was in an unstable condition; therefore, surgery and endoscopy of the sinus tissues and debridement were not possible. Despite antifungal and antibacterial therapy, the patient’s situation deteriorated, and the patient died on the evening of day 11 due to respiratory failure, hemodynamic instability, and mucormycosis with involvement of the orbit and probably the brain.

**Figure 3:**
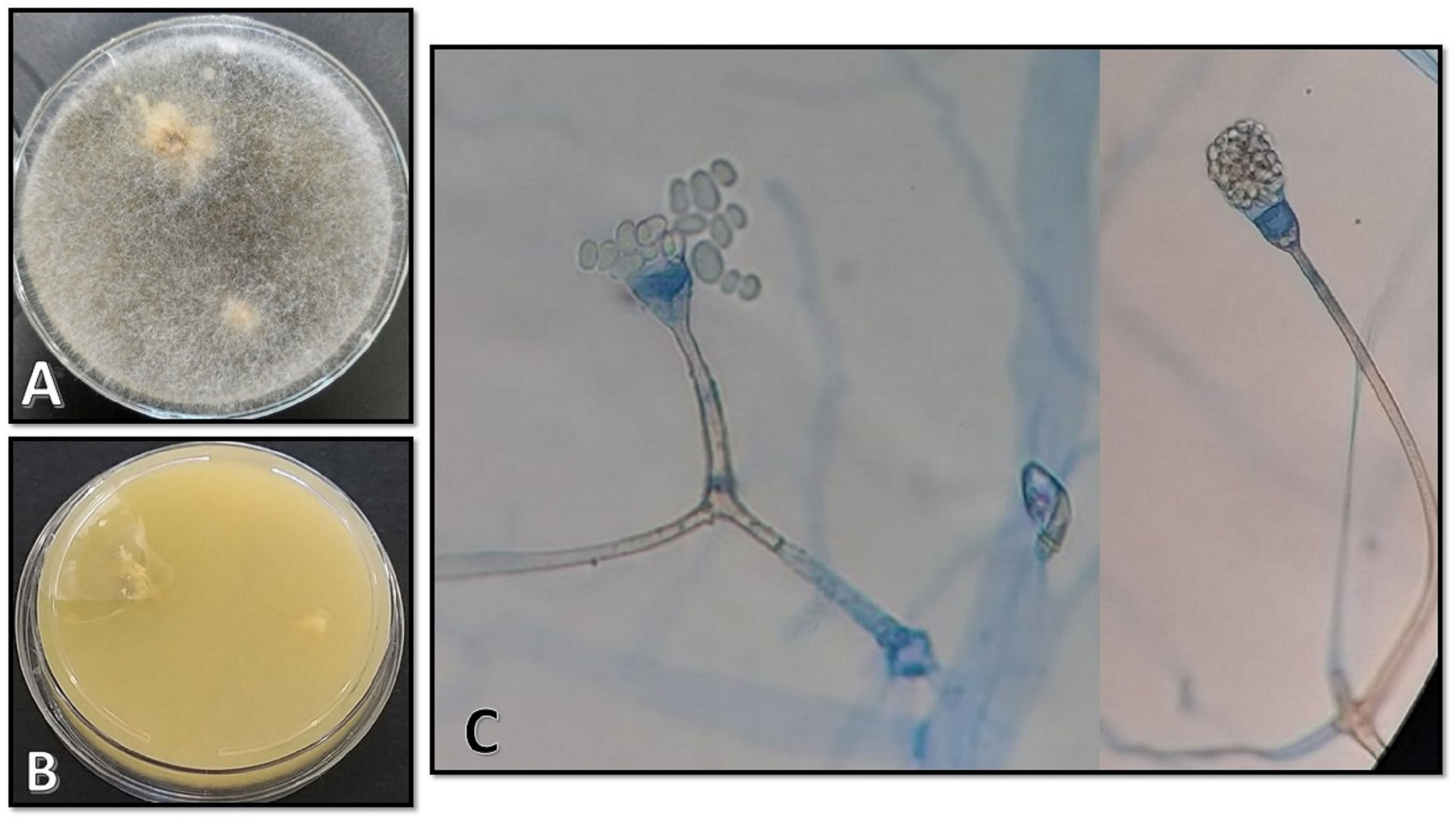
**(A, B)** Morphological characteristics of colonies on Sabouraud dextrose agar on the surface and the reverse after 4 days of incubation at 25°C. **(C)** Microscopic appearance of the isolate as observed on a slide prepared from the fungal culture.

The clinical samples obtained using sterile swabs and cultured on Sabouraud dextrose agar plates containing chloramphenicol (50 mg/L) (Biolife, Milan, Italy) yielded colonies of fluffy, cottony growth that filled the plate within 4 days. The colony surface was white when young, becoming cream-to-yellow or brownish-gray later on (**Figure 3A**). The reverse was white-to-pale yellow (**Figure 3B**). Microscopy of a slide prepared from the isolated colony revealed non-septate and branched hyphae, long and unbranched sporangiophores, and pear-shaped sporangia. Moreover, disintegration might have left a small collar at the foundation of the columella, and the sporangiospores were smooth and generally elongated and may have appeared as pale brown in mass (**Figure 3C**). Accurate morphologic identification at the species level was not possible; therefore, the isolate was subject to molecular identification. DNA was extracted using physical destruction of the cell wall by glass-bead manipulation followed by phenol–chloroform purification as described previously (Aboutalebian et al., 2021). The internal transcribed spacer (ITS) region was amplified using primer pairs ITS1 (TCCGTAGGTGAACCTGCGG) and ITS4 (TCCTCCGCTTATTGATATGC) (Aboutalebian et al., 2020). PCR products were purified and sequenced (Core Facilities Research Laboratory, Isfahan, Iran), and results were interpreted using NCBI BLAST (https://blast.ncbi.nlm.nih.gov/Blast.cgi) queries. The isolate was identified as *A. variabilis*, with a 99.86% sequence identity to the reference sequence. The sequence has been submitted to GenBank under the accession number OL804547.

Antifungal susceptibility testing (AFST) was performed based on the Clinical and Laboratory Standards Institute (CLSI) M38-A2 broth Dilution AFST (Wayne, 2008; Chakrabarti et al., 2010), and the minimum inhibitory concentrations were determined after 24 h of incubation at 35°C. *Candida parapsilosis* ATCC 22019 was used as quality control. AFST of the isolate against amphotericin B, fluconazole, itraconazole, voriconazole, posaconazole, and isavuconazole revealed minimum inhibitory concentration (MIC) values of 2, >64, 1, >16, 0.5, and 2 μg/ml, respectively, and minimum effective concentration (MEC) for caspofungin was >8 μg/ml. Written informed consent was obtained from the next of kin of the patient for the publication of any potentially identifiable images or data.

## Discussion

We report the first case of CAM caused by *A. variabilis* occurring in an apparently immunocompetent patient with severe COVID-19, with fungal identification obtained using sequencing of the ITS region of ribosomal DNA.

Mucormycosis is the third most common opportunistic invasive fungal infection and is characterized by high morbidity and mortality in immunocompromised and immunocompetent hosts (Bouza et al., 2006; Kontoyiannis et al., 2012). It manifests with a range of symptoms, such as rhino-orbito-cerebral, rhino-orbital, and pulmonary mucormycosis, which are caused by aspiration of the spores. The infection is potentially angioinvasive; therefore, the resulting disease is characterized by symptoms from vascular invasion and thrombosis, which lead to impaired blood flow and poor penetration of systemic antifungal treatment. The disease affects mainly immunocompromised individuals, including those with uncontrolled diabetes mellitus, hematologic malignancies, and high levels of iron and those on treatment with glucocorticoids. Mucormycosis can likewise appear as a cutaneous and subcutaneous illness in some trauma patients without known immunodeficiency.

*Rhizopus* is the genus most commonly involved in human mucormycosis, followed in frequency by *Mucor* and *Lichtheimia*, together representing 70%–80% of all cases of mucormycosis, whereas *Apophysomyces* spp. account for less than 3% of the infections (Gomes et al., 2011). Isolated from the environment of a mango orchard in northern India, the genus *Apophysomyces* was first described in 1979 (Misra and Lata, 1979). Until 2010, *Apophysomyces elegans* was regarded as the only member of this genus; however, the phylogenetic studies over the following years have revealed that *Apophysomyces* contains at least five species: *A. elegans*, *A. variabilis*, *A. trapeziformis*, *A. mexicanus* and *A. ossiformis*. Differences in apophysis and sporangiospores have been observed among the species (Alvarez et al., 2010). The variety of infections with *Apophysomyces* spp. is underappreciated, since these fungi do not usually grow on the standard fungal culture media utilized in clinical laboratories. These fungi require a special nutrient-deficient growth medium (e.g., Czapek agar), a high temperature in comparison to other human pathogens (37°C–42°C), and prolonged incubation (7–10 days) (Padhye and Ajello, 1988). In spite of their low virulence, *Apophysomyces* spp. are presently viewed as emerging pathogens amongst mucoralean fungi. Unlike other members of Mucorales that affect immunocompromised hosts and those with uncontrolled diabetes, *A. variabilis* mainly affects immunocompetent persons in the absence of underlying comorbidities (Ribes et al., 2000).

Recent studies have shown that CAM may occur especially in immunocompromised hosts (Pal et al., 2021). A number of risk factors for CAM have been recognized, and some of these were present in the current case. Our patient had a significant increase in CRP (108 mg/L), and elevated CRP levels and extreme neutropenia are essentially associated with worse survival (Cho et al., 2015). Gode et al. claimed that a CRP level >4 mg/dl would be related to poor prognosis in patients with acute invasive fungal rhinosinusitis (Gode et al., 2015). Similarly, Cho et al. likewise found that patients with a CRP level >5.5 mg/dl had worse outcomes (Cho et al., 2015). Twu et al. found that a CRP ≥1.025 mg/dl considerably accelerated the risk of developing orbital complications in patients with invasive fungal rhinosinusitis (Twu et al., 2021). In addition, Mucorales require iron to grow, and enhanced iron availability, including high ferritin levels, is a risk factor for CAM (Jose et al., 2021). Our patient had high ferritin levels during the period of infection, consequently precipitating the rapid growth of the fungus. Hyperglycemia in patients with COVID-19 because of previous diabetes mellitus, damage to beta cells in the pancreas by COVID-19, corticosteroid therapy, and stress-related increased cortisol levels with glucocorticoid therapy can damage phagocytic processes, failing to halt spore germination and growth and leading to CAM. Further endothelial damage and increased expression of endothelial receptors have been observed in patients with COVID-19. Therefore, in our patient, glucocorticoid treatment, hyperglycemia and its complications, and dysfunction of innate immune cells due to high-dose corticosteroid therapy and tocilizumab are probable contributors to the pathogenesis of CAM (Prakash et al., 2021).

*A. variabilis* has most often been isolated in cases of cutaneous mucormycosis (Chander et al., 2018). Nevertheless, a couple of cases of rhino-orbito-cerebral disease due to unusual Mucorales species have been reported; however, this is a condition that is probably underestimated due to difficulties in precise species identification in many laboratories. We reviewed 13 cases, including our current case, of ROCM published in the period 2000–2021, that were supported by data from molecular species identification. Eight of the 13 patients had been otherwise healthy, and only five of 13 were diabetic or had other underlying systemic diseases. The average age was 44.5 years (range, 24–74 years). As seen in **Table 1**, all these patients have been treated with liposomal amphotericin B or amphotericin B. Of the reviewed patients with a rhino-orbito-cerebral disease, six of 13 survived, and four died; for the remaining three patients, information was not available. Of the four who died, three were previously healthy, and one had diabetes (**Table 1**). Wolkow et al. reported a case of *A. variabilis* infection resulting in rhino-orbito-cerebral disease in an immunocompetent 74-year-old woman. This patient exhibited diffuse calvarial lytic lesions and overlying soft-tissue nodules, but without parenchymal intracranial involvement. There was radiographic and clinical evidence of infarction of the orbital contents and cavernous sinus thrombosis. That patient was treated with amphotericin B, isavuconazole, and terbinafine (Wolkow et al., 2017). Moreover, another case of *A. variabilis* infection has been reported, but no patient information is available (Bala et al., 2015).

**Table 1:** Clinical characteristics, treatment, and outcomes of patients with rhino-orbito-cerebral mucormycosis caused by rare or unusual species of Mucorales.

Case no.	Age/sex	Geography	Underlying condition	Causative agent of	Antifungal	Outcome and comments	References
1	59/M	Australia	Immunocompetent	*Apophysomyces elegans*	L-AmB	Cured	(Fairley et al., 2000)
2	24/M	Columbia	Immunocompetent	*A. elegans*	L-AmB	Cured	(Garcia-Covarrubias et al., 2001)
3	50/M	NA	Diabetes mellitus, allergic rhinitis, and previous sinusitis	*A. elegans*	L-AmB	Cured	(Liang et al., 2006)
4	31/M	India	Immunocompetent	*A. elegans*	AmB	Died	(Schütz et al., 2006)
5	56/F	India	Immunocompetent	*Saksenaea vasiformis*	AmB	Died	(Baradkar et al., 2008)
6	40/M	Malaysia	Immunocompetent	*S. vasiformis*	AmB	Cured	(Shatriah et al., 2012)
7	25/M	India	Immunocompetent	*A. elegans*	AmB	Cured	(Parsi et al., 2013)
8	NA/M	India	Diabetic	*Syncephalastrum racemosum*	NA	NA	(Mathuram et al., 2013)
9	45/F	India	Diabetic	*A. elegans*	L-AmB	NA	(Biswas et al., 2015)
10	NA	India	NA	*Apophysomyces variabilis*	NA	NA	(Bala et al., 2015)
11	74/F	USA	Immunocompetent	*A. variabilis*	L-AmB, ISC, TRF	Cured	(Wolkow et al., 2017)
12	46/M	Mexico	Diabetes	*A. ossiformis*	L-AmB	Died	(Martínez-Herrera et al., 2020)
13	40/F	Iran	Immunocompetent	*A. variabilis*	L-AmB	Died	This study

M, male; F, female; NA, not available; L-AmB, liposomal amphotericin B; AmB, amphotericin B; ISC, isavuconazole; TRF, terbinafine.

## Conclusions

This case alerts us to be more aware of opportunistic fungal infections caused by rare species of Mucorales in hospitals. Although it may be a difficult task, it is essential to be conscious of the correct detection and identification of microorganisms to improve the prophylaxis and therapeutic decision and outcome and to reduce mortality.

## Data availability statement

The raw data supporting the conclusions of this article will be made available by the authors, without undue reservation.

## Ethics statement

Ethical approval of the study was obtained from the ethics committee of Tehran University of Medical Sciences, Tehran, Iran (IR.TUMS.SPH.REC.1399.329). Written informed consent was obtained from the next of kin of the patient for the publication of any potentially identifiable images or data.

## Author contributions

ME and SA performed all the experiments and participated in data collection. SA drafted the manuscript and assisted in data analysis and interpretation. ME, MH, JH, AA, and SS participated in collecting the clinical isolate and in data collection. HM was in charge of supervising the study and critical review of the manuscript. All authors contributed to the article and approved the submitted version.

## Funding

This work was supported by Isfahan University of Medical Sciences, Isfahan, Iran (grant number 1400180), which we gratefully acknowledge. Also, the authors thank the staff at Shahid Beheshti Hospital, Kashan, Iran.

## Conflict of interest

The authors declare that the research was conducted in the absence of any commercial or financial relationships that could be construed as a potential conflict of interest.

## Publisher’s note

All claims expressed in this article are solely those of the authors and do not necessarily represent those of their affiliated organizations, or those of the publisher, the editors and the reviewers. Any product that may be evaluated in this article, or claim that may be made by its manufacturer, is not guaranteed or endorsed by the publisher.
